# Supplementary material for: Plasma Metabolites and Liver Composition of Broilers in Response to Dietary Ulva lactuca with Ulvan Lyase or a Commercial Enzyme Mixture
Source: Molecules. 2022 Nov 1;27(21):7425. doi: 10.3390/molecules27217425 (PMC9657063; doi:10.3390/molecules27217425)
Supplement: Supplementary file 1 [file molecules-27-07425-s001.zip › molecules-1943467-supplementary.pdf]

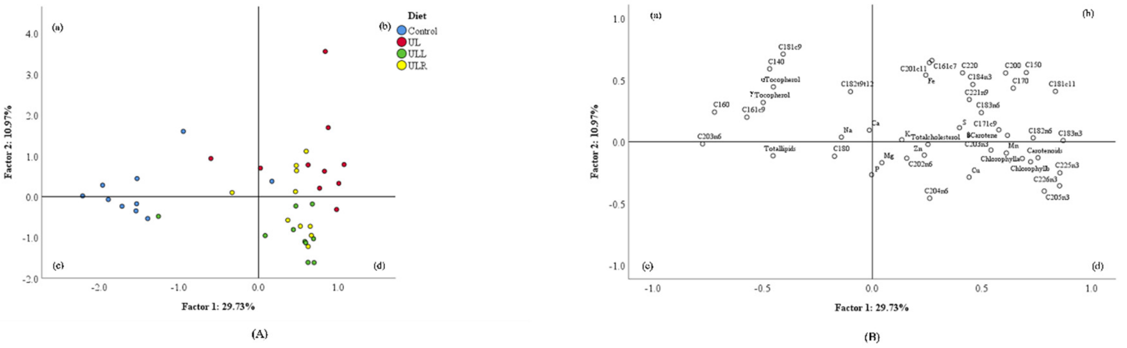

Figure S1: Loading plot of the first and second principal factors of the pooled data (A) and component score vectors (B) using hepatic metabolites from broilers fed with different diets: Control, corn-soybean based-diet; UL, based-diet plus 15% U. lactuca; ULR, UL diet with 0.005% commercial CAZyme (Rovabio® Excel AP); ULL, UL diet with 0.01% recombinant ulvan lyase.

Table S1. Chemical composition of seaweed *Ulva lactuca* and experimental diets (*n*=3) [18].

Item	*U. lactuca*	Control	UL	ULR	ULL
Energy, kcal ME†/kg as dry matter	2664	4649	4672	4607	4597
Proximate composition (g/100 g dry matter)					
Dry matter	88.7	89.0	89.2	89.3	89.4
Crude protein	28.2	23.3	23.1	22.8	23.3
Crude fat	2.85	8.81	9.00	9.75	10.3
Ash	31.7	6.58	8.83	8.77	8.86
Fatty acid profile (g/100 g of total fatty acids)					
14:0	0.61	0.01	0.13	0.11	0.11
16:0	33.6	9.86	9.88	9.96	9.87
16:1*c*9	4.17	0.15	0.27	0.25	0.25
17:0	0.58	0.06	0.04	0.05	0.05
17:1*c*9	0.96	0.03	0.03	0.03	0.03
18:0	1.26	3.17	3.14	3.20	3.21
18:1*c*9	14.9	29.6	28.7	28.5	27.9
18:2*n*-6	6.14	53.5	52.5	52.7	53.4
18:3*n*-3	17.3	1.15	1.41	1.46	1.38
18:4*n*-3	12.8	n.d.	0.73	0.71	0.66
20:0	0.34	0.37	0.37	0.37	0.37
20:4*n*-6	0.96	n.d.	0.02	0.03	0.03
20:5*n*-3	0.90	0.03	0.04	0.04	0.04
22:0	1.54	0.61	0.64	0.63	0.67
Diterpene profile (µg/100 g)					
α-Tocopherol	79.3	254	254	234	224
α-Tocotrienol	n.d.#	5.00	3.26	3.56	2.77
β-Tocopherol	n.d.	0.72	0.77	0.74	0.72
γ-Tocopherol+β-Tocotrienol	n.d.	5.47	4.15	4.18	3.57
γ-Tocotrienol	n.d.	6.18	4.09	4.54	3.46
δ-Tocopherol	n.d.	0.96	0.69	0.73	0.70
Pigments (mg/100 g)					
β-Carotene	170	1.28	33.6	28.3	28.4
Chlorophyll *a*[1]	2311	5.78	599	589	588
Chlorophyll *b*[2]	1666	8.46	438	424	428
Total Carotenoids[3]	510	6.04	145	144	134

Mineral profile (mg/kg dry matter)					
Bromine	694	4.10	211	157	177
Calcium	6202	20263	11315	11195	11756
Copper	3.73	17.8	16.4	19.1	19.7
Iodine	45.1	1.09	14.0	11.2	11.7
Iron	537	296	343	310	326
Magnesium	25889	2723	7826	7913	8189
Manganese	39.0	154	128	128	139
Phosphorous	2786	10728	8941	8918	9284
Potassium	38822	15850	21079	21462	21639
Sodium	52133	3388	13727	13924	15122
Sulphur	49265	3379	14423	14670	15098
Zinc	8.96	121	100	113	107

Diets: Control, corn-soybean based-diet; UL, based-diet plus 15% *U. lactuca*; ULR, UL diet with 0.005% commercial CAZyme (Rovabio® Excel AP); ULL, UL diet with 0.01% recombinant ulvan lyase.

[†] ME, metabolized energy; [#]Co-eluted with α-tocopherol; n.d., not detected.

[1] Ca = 11.24 A662 - 2.04 A645.

[2] Cb = 20.13 A645 - 4.19 A662.

[3] Ca+b = 7.05 A662 + 18.09 A645.

18. Costa, M.M.; Pestana, J.M.; Carvalho, P.; Alfaia, C.M.; Martins, C.F.; Carvalho, D.; Mourato, M.; Gueifão, S.; Delgado, I.; Coelho, I.; Lemos, J.P.C.; Lordelo, M.M.; Prates, J.A.M. Effect on Broiler Production Performance and Meat Quality of Feeding *Ulva lactuca* Supplemented with Carbohydrases. *Animals* **2022**, *12*, 1720. https://doi.org/10.3390/ani12131720
